# Signatures of Adaptation in Mitochondrial Genomes of Palearctic Subterranean Voles (Arvicolinae, Rodentia)

**DOI:** 10.3390/genes12121945

**Published:** 2021-12-02

**Authors:** Olga Bondareva, Evgeny Genelt-Yanovskiy, Tatyana Petrova, Semen Bodrov, Antonina Smorkatcheva, Natalia Abramson

**Affiliations:** 1 Laboratory of Evolutionary Genomics and Paleogenomics, Zoological Institute RAS, 199034 Saint-Petersburg, Russia; genelt.yanovskiy@gmail.com (E.G.-Y.); p.tashka@inbox.ru (T.P.); BodrovS@gmail.com (S.B.); 2 Department of Vertebrate Zoology, Biology Faculty, Saint-Petersburg State University, 199034 Saint-Petersburg, Russia; tonyas1965@mail.ru

**Keywords:** selective pressures, mitochondrial protein-coding genes, subterranean voles, adaptations, subterranean lifestyle

## Abstract

This study evaluates signatures of selection in the evolution of the mitochondrial DNA of voles, subfamily Arvicolinae, during the colonization of subterranean environments. The comparative sequence analysis of mitochondrial protein-coding genes of eight subterranean vole species (*Prometheomys schaposchnikowi*, three species of the genus *Ellobius*: *Ellobius talpinus*, *Ellobius fuscocapillus* and *Ellobius lutescens*, two species of the genus *Terricola*: *Terricola subterraneus* and *Terricola daghestanicus*, *Lasiopodomys mandarinus,* and *Hyperacrius fertilis*) and their closest aboveground relatives was applied using codon-substitution models. The highest number of selection signatures was detected in genes *ATP8* and *CYTB.* The relaxation of selection was observed in most mitochondrial DNA protein-coding genes for subterranean species. The largest amount of relaxed genes is discovered in mole voles (genus *Ellobius*). The number of selection signatures was found to be independent of the evolutionary age of the lineage but fits the degree of specialization to the subterranean niche. The common trends of selective pressures were observed among the evolutionary ancient and highly specialized subterranean rodent families and phylogenetically young lineages of voles. It suggests that the signatures of adaptation in individual mitochondrial protein-coding genes associated with the colonization of the subterranean niche may appear within a rather short evolutionary timespan.

## 1. Introduction

Being among the most diverse groups of mammals, rodents have colonized nearly all major terrestrial habitats around the world. Most species dwell on the surface, yet many species are able to dig complex tunnels where they nest, rest, and shelter from predators. These forms are commonly classified as fossorial, i.e., they dig burrows and tunnels yet spend only a part of their active time there. Some are nearly completely subterranean—they build networks of underground corridors, where most of their activities, including foraging, take place, and therefore they are referred to as “truly” subterranean [1]. There are more than 250 species of fossorial and subterranean rodents (combined in 38 genera, six families), that are distributed across all continents except Australia and Antarctica [1].

Subterranean rodents represent a powerful study system for testing hypotheses about adaptive evolution during the colonization of a novel and highly contrasting habitat compared to aboveground species. They inhabit an environment characterized by high levels of carbon dioxide, low levels of oxygen, high humidity, and relatively constant temperature [2].

Voles and lemmings, subfamily Arvicolinae, are the youngest rodent group that emerged via the fastest documented adaptive radiation among recent mammals [3,4]. The number of extant arvicoline rodents is eight times greater than in the sister subfamily Cricetinae; the most recent common ancestors of both are known in the fossil record from the Late Miocene, ca 10 Ma [5]. The earliest arvicolines are known from the Late Miocene both in Eurasia and North America [5,6] and they currently inhabit almost all landscapes and habitats in the Northern Hemisphere. Arvicolinae provide a convenient opportunity for testing various hypotheses about the tempo and mode of adaptive evolution, including those concerned with the evolution towards fossoriality, with a comparative method. The members of this phylogenetically young group display a full spectrum of substrate utilization patterns from surface-dwelling to even arboreal form (e.g., tree voles, *Arborimus* Taylor, 1915 [7,8]) to highly specialized subterranean species (*Ellobius* Fischer, 1814 and *Prometheomys* Satunin, 1901 [9]).

The mitochondrial genome has historically been thought to evolve neutrally, thanks in large part to the dominance of Kimura neutral theory [10], and protein-coding genes (e.g., *CYTB*, *COX1*) for a long time have been used in phylogenetic reconstructions of various taxonomic groups [11,12]. The assumption of selective neutrality of mitochondrial DNA is simplistic since mitochondrial protein-coding genes are involved in oxidative phosphorylation and responsible for producing up to 95% of the energy of eukaryotic cells. Due to the importance of this biochemical pathway, evaluating selective pressures acting on mitochondrial proteins could provide key insight into the adaptive evolution of the mitochondrial genome as has been suggested by recent studies [13,14]. Signals of positive selection related to shifts in ecological specialization and changes in metabolic requirements have been detected in various organisms from anthropoids [15,16] and fish [17] to different mammals and human populations [18,19,20,21,22].

Following the introduction of computationally tractable codon substitution models [23,24] nearly two decades ago, there has been a persistent interest in using these models to study the past action of natural selection on protein-coding genes. Positive selection can be inferred as the estimated ratio (ω) of non-synonymous (*dN*) to synonymous (*dS*) substitution [25,26]. Using improved evaluation approaches, molecular research on species with altered energy expenditures shows the adaptability of the mitochondrial genome. For instance, birds and bats require a metabolic rate which is 3–5 times greater than the maximum observed in actively moving terrestrial mammals of similar size [27,28]. Evaluation of selection signals in the mitochondrial genes of bats showed that the rate of nonsynonymous substitutions exceeds the rate of synonymous ones in genes *ND2*, *ND3*, *ND4L*, *ND4*, *ND5*, *ND6*, and *COX3* and, therefore, the value ω is significantly greater than in other mammals [21]. Thus, numerous studies performed on various vertebrates and invertebrates convincingly show that the variability of mitochondrial genes may have adaptive significance [18,19,29,30,31,32].

A recent study by Da Silva et al. [33] found a significantly greater ω in the *CYTB* gene among independent lineages of subterranean rodents (tuco tucos, coruros, pocket gophers, and mole rats) as compared to their aboveground relatives, suggesting a potential connection between directional selection of this gene and the niche shift to being subterranean. Research on subterranean representatives *Ctenomys* Blainville, 1826 (Ctenomyidae) and *Spalacopus* Wagler, 1832 (Octodontidae) and showed the presence of positive selection in all mitochondrial genes except the *ND3* gene. It showed that habitat associated with the colonization of the subterranean niche, especially the transition from oxygen-rich to hypoxic atmospheres had led to the changes in the selective regimes of proteins involved in respiration. Our previous study [34] showed the relaxation of selection at the mitochondrial *CYTB* gene in Arvicolinae subterranean species by several computational approaches.

Signatures of selection were recently found in several mitochondrial genes of subterranean rodents [22,33] using the phylogenetic framework at the family level and over a vast evolutionary period. Here, we applied a similar approach in search for selection traces at a significantly smaller evolutionary timescale and within a lower taxonomic level: several subterranean species of the Arvicolinae subfamily. We aimed to determine whether this fast transition left signatures of selection in mitochondrial protein-coding genes and whether the substitutions occur at homologous or different sites and genes, comparing different genera within a subfamily and different species within one genus that have independently transitioned to a subterranean ecology. To this end, we focused on evaluating the selection signals in mitochondrial genomes of the subterranean Palearctic voles on the background of their closest aboveground relatives using comparative sequence analysis. By considering complete mitochondrial genome sequences, we evaluate the magnitude of selective pressures on mitochondrial genes that occurred on the branches leading to subterranean Arvicolinae. We tried to answer the following questions: (1) is the relaxation of selection observed by us previously in the analysis of the *CYTB* gene also traceable and uniformly distributed among the rest of the mitochondrial genome; (2) does the shift to a subterranean niche causes changes in the same genes and the same amino acids or do different genes behave differently in phylogenetically independent lineages; (3) are the trends of a consistent selective pressure on the mitogenome repeated during the transition to subterranean ecology in the studied taxa; and (4) do selective signals occur in the mitochondrial genomes of the phylogenetically young subterranean vole lineages and ancient and highly specialized subterranean rodent families in homologous genes and sites?

## 2. Materials and Methods

### 2.1. Taxa Selection and Samples

In total, we analyzed 61 complete published mitochondrial genomes of representatives of Arvicolinae and Cricetinae subfamilies (Supplementary Table S1). Hereinafter, we use the taxonomic classification following Gromov and Polyakov [35], Musser and Carleton [36], and Abramson and Lissovsky [37]. 

For the purposes of this study, we categorized species as subterranean or aboveground based on their behavioral habits rather than morphological traits. A species was considered subterranean if it is known to conduct the majority of their life underground, perform regular digging activities, and their aboveground excursions are limited to the vicinity of burrow openings [38,39]. Within our dataset, eight species in five genera fit these definitions: *E. talpinus* Pallas, 1770; *E. lutescens* Thomas, 1897; *E. fuscocapillus* Blyth, 1843; *T. subterraneus* de Selys-Longshamps, 1836; *T. daghestanicus* Shidlovsky, 1919; *L.s mandarinus* Milne-Edwards, 1871; *H.s fertilis* True, 1894; and *P.s schaposchnikowi* Satunin, 1901.

Two arvicoline genera considered as “truly subterranean” are *Ellobius* Fischer, 1814 (mole voles) and *Prometheomys* Satunin, 1901 (the long-claw vole) [1,2]. Not only does all of their activity take place underground but they demonstrate a set of obvious morphological and life-history modifications associated with this lifestyle. Both *Ellobius* and *Prometheomys* have short soft pelage, small/tiny eyes, and reduced ears. Moreover, mole voles have a highly modified skull with prominent extrabuccal incisors which are the characteristic of chisel-tooth diggers [9,40], whereas the long-claw vole is a specialized scratch digger [41]. In *Terricola* Fatio, 1867 voles, the tendency towards fossorial mode of life is thought to result in short dense fur, small eyes, short pinnae hidden in the fur, and the characteristics of slow life history [42,43,44,45,46]. Another fossorial arvicoline, the mandarin vole *L. mandarinus*, does not seem to display marked morphological adaptations that would facilitate digging and has been proposed to decrease the costs of forage tunnel construction due to living in cooperative family groups [39,47]. The last one, Kashmir vole *H. fertilis,* is poorly studied but has been classified as subterranean by Begall et al. [1]. *Arvicola amphibius* Linnaeus, 1758 is often listed among subterranean forms because within this species both semi aquatic and subterranean populations exist. However, the sample we sequenced and included in the analysis is from the semi aquatic population, so we referred it to the background branch.

**Figure 1:**
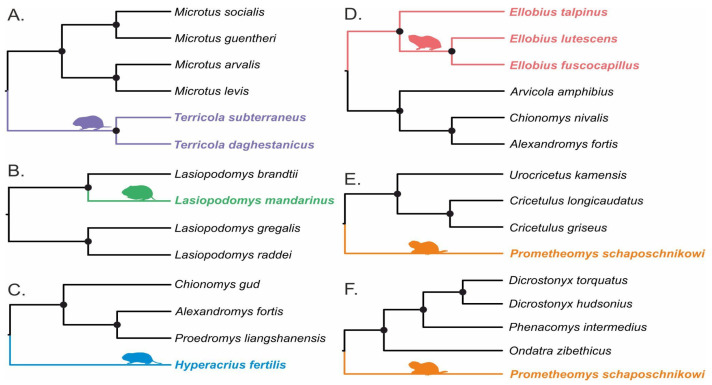
Trees used for tracing signatures of the selection pressures on the independent subterranean vole species. Separate trees show the phylogenetic relationship of subterranean voles (colored) and their closest aboveground relatives: *Terricola* species (**A**), *L. mandarinus* (**B**), *H. fertilis* (**C**), *Ellobius* species (**D**), and *P. schaposchnikowi* (**E**) comparing with aboveground representatives of Cricetinae subfamily, and (**F**) comparing with aboveground representatives of Arvicolinae subfamily, 1st radiation). Fifty-percent majority rule consensus trees from Bayesian inference analysis are given. Black circles show nodes with Bayesian posterior probabilities above 0.99.

For a more detailed analysis of individual subterranean species, we additionally divided them into groups. The subdivision was carried out according to the principle of phylogenetic nearest aboveground taxa selection (Figure 1). The phylogenetic nearest taxa for *H. fertilis* were selected according to its specified phylogenetic position in the tribe Arvicolini [48]. Two different sets were chosen for *P. schaposchnikowi*, considering its monotypy and basal position within the subfamily Arvicolinae. Thus, we first tested the *Prometheomys* against the subfamily Cricetinae (hamsters) which is a sister clade to the Arvicolinae (Group E). Second, we tested it against several representatives of the Arvicolinae (Group F) that constitute the so-called first radiation within the subfamily.

### 2.2. Alignment

The sequences of complete mitochondrial genomes were aligned using the Mauve 1.1.1 [49] implemented as the Geneious Prime 2019.1 plugin (https://www.geneious.com, accessed on 26 November 2021). The concatenated sequence of 13 protein-coding mitochondrial genes was separately aligned using MAFFT 7.222 [50].

### 2.3. Phylogenetic Reconstructions

Phylogenetic reconstructions were carried out in MrBayes 3.2.2 [51] using either seven nuclear genes (6421 bp) (see methods in [34]) or 13 protein-coding mitochondrial genes (11,391 bp) in the case of *H. fertilis* (since nuclear genes are not available for this species). At this taxonomic level, the topology did not differ depending on nuclear or mitochondrial data on which the phylogenetic reconstruction was built. 

The following analysis parameters were set: nst = mixed and the γ distribution of the rates of substitutions between sites, the division into partitions by genes was used. Each analysis started with a random tree and had two replicates with four Markov chains (MCMC) and 1 million generations, with the results recorded every hundredth generation. Stationarity and convergence of separate runs were assessed using ESS statistics in Tracer v1.6 [52]. The trees were visualized using the FigTree v1.6 program (http://tree.bio.ed.ac.uk/software/figtree/, accessed on 26 November 2021).

### 2.4. Base Mitochondrial Statistics Analysis

The base nucleotide composition was calculated in Geneious Prime 2019.1 (https://www.geneious.com, accessed on 26 November 2021) for all subdivided groups (Figure 1). The displacement in the nucleotide composition (GC-skew) was studied by calculating the relative frequencies of nucleotides C and G [53,54] using the BioSeqUtils package in BioPython [55] in Python 3.0. The difference in the mean values was checked using the unpaired Wilcoxon test in the R software v.3.4.4 [56]. The mitochondrial genome of *H. fertilis* was excluded from this analysis because of its incompleteness [57] and the possibility of introducing errors into the analysis.

### 2.5. Convergent and Parallel Amino Acids Substitution Detection

The evaluation of both convergent and parallel amino acid substitutions was carried out by the ProtParCon program [57] with a further search for amino acids characteristic only of subterranean rodents: We looked for replacements occurring in at least three subterranean taxa using a custom script on Python 3. The significance of the substitution frequency was estimated with the ProtParCon subfunction using additionally Holm multiple adjustments in R software v.3.4.4.

### 2.6. Analysis of Selective Pressures

Selective pressures were estimated as variation in the levels of non-synonymous (*dN*) and synonymous (*dS*) substitutions as well as ω using several approaches. We apply three: classic codeml program [58,59] with the comparisons between free-branch and M0, and free-branch and neutral-branch models; improved branch-site model-aBSREL (an adaptive branch-site REL test for episodic diversification [60]) and RELAX [61] to test whether the strength of natural selection has been relaxed or intensified along a specified set of branches. In addition, we search sites under positive selection with MEME (Mixed Effects Model of Evolution, [62]). All programs except codeml are implemented in the DataMonkey webserver (datamonkey.org accessed on 26 November 2021, [63,64]).

We used a full set of mitochondrial protein-coding genes (*n* = 13). First, we used a concatenation of protein-coding mitochondrial genes to determine selection pressure across all Arvicolinae subfamily subterranean rodents simultaneously. As a phylogenetic topology, we used the tree published earlier [48]. Further, the total set of species was subdivided into several subsets so that each subset contained a subterranean species and its phylogenetically closest or sister aboveground species (Figure 1) for a more detailed analysis. The estimation for *P. schaposchnikowi* was assessed twice, in respect to hamsters (Figure 1E) and to other representatives of the Arvicolinae (Figure 1F) due to its basal position within the subfamily. In this case, the calculation was carried out independently for each mitochondrial gene. The calculated *p*-values of all likelihood-ratio tests (LRTs) were corrected using Holm multiple adjustments in R software v.3.4.4. The difference in the mean values for the free ratio model was checked using the unpaired Wilcoxon test also in the R software v.3.4.4.

The classical codeml program [58,59] was used with the ete-toolkit interface [65]. A branch model assumes significant differences between ω values for marked branches (foreground or ωfrg) and the rest branches of the tree (background branches or ωbkg). The branch model-based approach allows estimation of selection level at an individual subterranean species (colored on Figure 1) compared only with phylogenetically close aboveground taxa (marked black on Figure 1), or at all subterranean species simultaneously. In all the analyses, subterranean species were marked as foreground and aboveground taxa as background branches. For each subterranean species (or group of species for *Ellobius* and *Terricola*) and with the complex analysis of all subterranean rodents at the same time, analysis was implemented with a free-branch model (b_free, where ωfrg and ωbkg are free), a neutral-branch model (b_neut, where ωfrg is fixed to one) and an M0 model, where all branches evolve at the same rate. LRT was calculated to compare different models. The comparison between free-branch and M0 shows whether foreground branches have ω significantly different from the rest of the tree. Additionally, the comparison between free-branch and neutral-branch models detects if the value of ωfrg was significantly greater than 1. The values 999 and 0.001 were regarded as errors.

The aBSREL differs from other branch-site model implementation by inferring the optimal number of ω classes for each branch. After aBSREL fits the full adaptive model, the LRT is performed at each branch and compares the full model to a null model where branches are not allowed to have rate classes of ω > 1. RELAX was employed to test changes in the intensity of selection (relaxation or intensification) in phylogenetic branches. A significant K > 1 estimate indicates that selection strength has been intensified along the test branches, while a significant K < 1 indicates that selection strength has been relaxed along the test branches. MEME employs a mixed-effects maximum likelihood approach to test the hypothesis that individual sites have been subjected to episodic positive or diversifying selection. Thus, MEME aims to detect sites evolving under positive selection under a proportion of branches. We changed the confidence level manually from 0.1 to 0.05 for more significant results.

## 3. Results

### 3.1. Comparison of Base Nucleotide Composition and Gene Order

**Figure 2:**
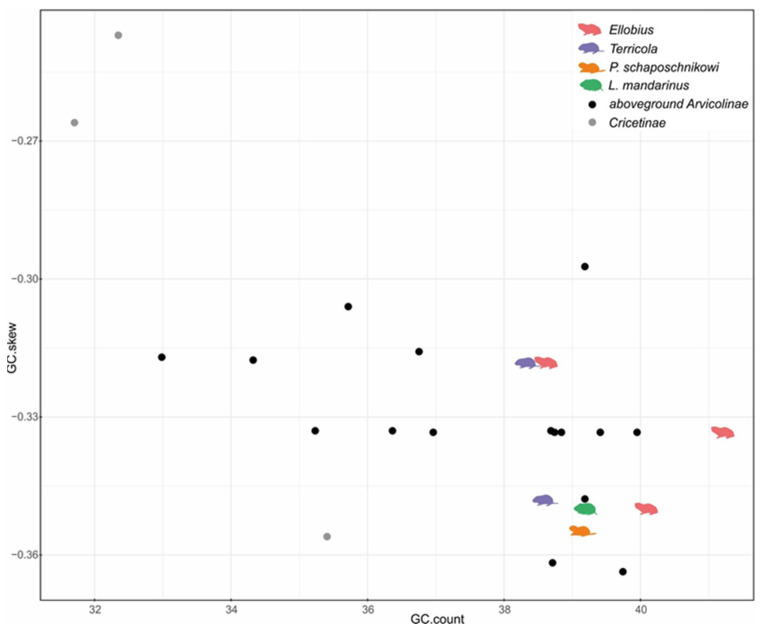
Variation of GC-content and GC-skew values in mitochondrial genomes of Arvicolinae. Black dots—aboveground vole species (Arvicolinae), grey dots—hamsters (Cricetinae), color symbols—subterranean vole species.

Variation in GC-content among mitochondrial genomes of subterranean species was lower than among aboveground species (Figure 2). There were no differences in mean values of GC-content and GC-skew. Gene order among all studied mitochondrial genomes remained unchanged.

### 3.2. Selection Relaxation or Intensification

We observe a tendency towards relaxation of selection in concatenated mitochondrial protein-coding genes of subterranean rodents in both branch and free ratio codeml analyses for the entire Arvicolinae phylogenetic tree [48]. Thus, observed *dN*/*dS* values for all subterranean rodents is significantly higher than for aboveground Arvicolinae, but remains strongly less than one (Table 1) based on branch analysis results. The average ω-values in the free ratio analysis constitute 0.091 and 0.055 for subterranean and aboveground, respectively. This analysis was carried out with no a priori designation of subterranean lineages and showed significant differences between mean values by unpaired Wilcoxon test comparison (*p*-value = 0.012).

Significant selection signs were detected in three separately analyzed subterranean taxa using the ete-evol workflow based on codeml (Table 1, for all data see Supplementary Table S2). According to this analysis, eight mitochondrial protein-coding genes in Ellobius show significantly greater *dN*/*dS* values compared to the background taxa of Arvicola amphibius, Chionomys nivalis Martins, 1842 and Alexandromys fortis Büchner, 1889. Two genes (*COX3* and *CYTB*) were detected as genes with greater ω in *L. mandarinus* compared to other species of genus Lasiopodomys. Only *COX3* demonstrated signatures of selection in *P. schaposchnikowi* on the background of Ondatra zibethicus Linnaeus, 1766, true and collared lemmings (Table 1). Greater *dN*/*dS* values observed in foreground branches in all above mentioned comparison pairs indicated signatures of relaxed selection in mitochondrial protein-coding genes of subterranean rodents (Table 1, Supplementary Table S2). Since all the ω values we obtained are less than one, we can only talk about the weakening of the purifying selection in the case of this analysis.

All genes in *Ellobius* except *ATP8* showed signatures of non-neutral selection together with all 13 mitochondrial protein-coding genes of two Terricola species and seven genes of *L. mandarinus* (Supplementary Table S2).

The aBSREL approach also found evidence of episodic positive selection in analyzed subterranean rodents (Figure 1). It is observed in *COX2* for *E. lutescens* (but not in other species of the genus *Ellobius*) and in two genes for *P. schaposchnikowi*: *ATP8* as compared with *Arvicolinae* species (Group F) and *ND5* with respect to hamsters (Group E) (Table 2).

The RELAX analysis showed changes in the intensity of purifying selection in mitochondrial protein-coding genes both for all subterranean species together and for three separate subterranean species compared with aboveground ones (Table 3 and Table S3). Eight genes with significant K-values < 1 and traces of selection relaxation were detected in *Ellobius*: *ATP6*, *COX1*, *COX3*, *CYTB*, *ND1*, *ND2*, *ND3*, and *ND4*. Five genes (*COX1*, *COX3*, *ND2*, *ND4*, and *ND5*) were under relaxed selection in *P. schaposchnikowi* compared to Group F (Figure 1). Selective pressure was also significantly weakened in the *COX3* gene of *P. schaposchnikowi* compared to Cricetulus species (Group E). Analysis indicated three genes with signatures of relaxed selection in *L. mandarinus*: *COX1*, *COX3*, and *CYTB* (Table 3). No genes with a significant relaxation or intensification of selection were found for the remaining subterranean voles: *H. fertilis* and both investigated *Terricola* species.

A number of orthologous genes showed relaxation of selection in several subterranean species. For instance, *COX1* and *COX3* genes demonstrated relaxation in all three Ellobius species, *P. schaposchnikowi*, and *L. mandarinus*. Some genes were detected under relaxed selection in *Ellobius* species and *P. schaposchnikowi* (ND2, ND4) or *Ellobius* species and *L. mandarinus* (CYTB). 

### 3.3. Estimation of Selective Pressures in Sites of Protein-Coding Genes

We applied the MEME approach for each subterranean taxon individually (Figure 1) to search for sites under positive selection in all mitochondrial protein-coding genes. Results of this analysis are shown in Table 4.

Various sites under positive selection have been found in the orthologous mitochondrial genes. The intensity and evidence of positive selection differs in several genes: CYTB, ND4, and ND5 have the greatest number of sites under positive selection, while no sites at all were detected in the COX3 gene. The largest number of sites under positive selection were found for *P. schaposchnikowi*, Ellobius group, and *L. mandarinus*. In genes ATP6, COX2, ND1, and ND3 we found sites only for *L. mandarinus* and in gene ND4L only for the Ellobius group. Three sites in two genes (ATP8 and CYTB) were detected for *H. fertilis*. Homologous nucleotide site 434 in COX1 gene was detected as being under positive selection both for the *Ellobius* species and *P. schaposchnikowi*. No sites under positive selection were traced for two Terricola species.

### 3.4. Search for Parallel and Convergent Amino Acid Substitutions

Using ProtParCon, we found six amino acid substitutions meeting our search criteria: COX1 Met73Ile, COX3 Ile121Val, ND5 Phe446Leu, CYTB Thr56Ser, CYTB Ile338Val, and CYTB Ala357Thr (Supplementary Table S4). However, all of them appeared to be insignificant upon further verification.

## 4. Discussion

Signatures of selection were recently found in several mitochondrial genes of subterranean rodents [22,33,66,67] using the phylogenetic framework at the level of families that evolved over a vast evolutionary period. Here, we applied a similar approach in search for selection traces at a significantly smaller evolutionary timescale and within a lower taxonomic level: several subterranean species of the Arvicolinae subfamily. Applying various approaches in searching selection signatures in Arvicolinae mitochondrial protein-coding genes, we have found (1) genes with signs of selection relaxation in subterranean species; (2) among subterranean species the number of genes with selection signatures substantially differs. Signatures of selection relaxation were identified in both series of analyses implemented for each subterranean species separately and on the complete dataset of the Arvicolinae subfamily.

**Figure 3:**
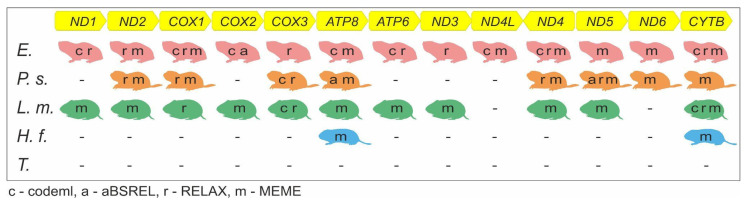
Genes with significant changes in selective pressures for each of the subterranean species. Letters inside each silhouette indicate the analyses in which a significant result was obtained. *E*—*Ellobius species*, *P.s.*—*P. schaposchnikowi*, *L.m.*—*L. mandarinus*, *H.f.*—*H. fertilis*, *T.*—*Terricola* species.

Gene *COX3* demonstrated greater ω values in codeml branch analysis for both *P. schaposchnikowi* and *L. mandarinus* and *CYTB* for *L. mandarinus* and *Ellobius* species relative to sister aboveground taxa. According to the RELAX analysis, *COX1, COX3, ND2,* and *ND4* show selection relaxation for at least two subterranean species (Figure 3). A list of common genes with sites under positive selection discovered in branch-site MEME analysis includes more than half of the mitochondrial protein-coding genes: *ATP8, COX1, CYTB, ND2, ND4, ND5,* and *ND6*. Genes *COX1* and *COX3* were found to be the most affected by selection pressure according to the results of several approaches based on complete gene sequence analysis, i.e., excluding MEME results (Figure 3).

Similar results were obtained by Da Silva et al. [33], indicating a significant difference for ω values in *CYTB* sequences of subterranean rodents from families Ctenomyidae, Geomyidae, and Bathyergidae compared to their aboveground closely related species. Significantly greater ω ratio in subterranean groups relative to aboveground ones was shown also by Tomasco and Lessa [22]. Moreover, the distribution of non-synonymous mutations indicated the considerable changes in *CYTB* of animals, which were confronted with more severe hypoxia because of higher altitude and a colder, drier climate and were facing a higher purifying selection pressure [68]. Finally, Tomasco and Lessa [66] found increased values of ω in *COX2* of subterranean coruros *Spalacopus* (almost 30×) and tuco-tucos *Ctenomys* (11×) relative to aboveground octodontoids. Our previous studies on the Arvicolinae subfamily also showed the relaxation of selection strength in *CYTB* reflecting an increased ratio (ω) in subterranean lineages compared to non-subterranean lineages [34]. Later, indicated changes were demonstrated for all mitochondrial protein-coding genes. Subterranean species south American tuco-tucos and the related coruro in the work of Tomasco and Lessa [66] showed a significantly greater ω compared to their aboveground counterparts in 11 of 13 mitochondrial genes. Convergent changes were also found between the studied subterranean genera and other mammals adapted to hypoxia. All genes except *ND3* show amino acid properties consistent with positive destabilizing selection. Proteins varied in the fraction of strong positively selected amino acid properties per site, from 0.03 in *COX3* to 0.38 in *ATP8*. In most genes one or several (for *ND4L*, *ND5,* and *COX1*) positively selected sites were detected. Tavares and Seuánez [67] showed significant selection relaxation in most mitochondrial protein-coding genes of subterranean hystricomorphs (African mole-rats, tuco-tucos, and coruro). Conversely, selection intensification was found in three genes in fossorial sciuromorphs (ground squirrels, chipmunks, and marmot). Thus, summing up this brief comparison with other studies of selective pressures in mitochondrial genomes of subterranean rodents, we observe the common trends in evolutionary old and highly specialized subterranean rodent families and phylogenetically young lineages of voles.

According to some assumptions [2], such an increase in the level of selection in mitochondrial protein-coding genes could be a consequence of the low effective population size of subterranean rodents. When ω values are smaller than one, the study of a single gene does not allow us to reject alternative, non-selectionist explanations of rate variation, such as a variation in metabolic rate, body mass, population size, and generation time among lineages [69,70,71]. However, under a relaxation of a purifying selection model, the same pattern of variation among lineages is expected in all genes. In contrast, our study found (a) most, but not all genes have greater ω in subterranean lineages; and (b) different combinations of genes with selection signatures are detected by approaches used in studied species.

We found that some genes with an increased number of selection signatures appear in various analyses more frequently than others. It may be supposed that the number of selection signatures is partly positively correlated with the rate of gene evolution. Mutation rates between mitochondrial gene families are distributed as follows: *ATP* > *ND* > *CYTB* > *COX* according to Lopez [72]. In accordance with this assumption our results showed that both *ATP* genes display an increase in signatures of selection in subterranean species, except in *T. subterraneus* and *T. daghestanicus*. Moreover, selective traces in genes from the *ATP*-group are detected in several analyses: RELAX (*ATP6* for genus *Ellobius*), and aBSREL (*ATP8* for *P. schaposchnikowi*). *ATP8* demonstrated positively selected sites in almost all analyzed species except *Terricola* species, and, thus, it is one of the two genes in our dataset with the most pronounced selection signs (Figure 3). Genes of the *ND* group vary in the degree of selective pressures. Genes *ND2*, *ND4,* and *ND5* were detected in all of the performed analyses in three out of five analyzed subterranean species. Aside from positively selected sites detected by MEME, significant changes in selective pressure were found in *ND1, ND2, ND4, ND4L,* and *ND5* genes (Figure 3). The *CYTB* gene shows a significant difference in ω levels between subterranean and aboveground species and confirmed selection relaxation for *Ellobius* species and *L. mandarinus*. These results reproduce our earlier findings performed in the analysis of the *CYTB* alone and with a different set of compared species [34]. In addition to those previously reported, we have now detected positively selected sites for *P. schaposchnikowi*, *Ellobius* species, *L. mandarinus,* and *H. fertilis*. Thus, *CYTB* on a par with *ATP8* appears as the gene with maximum selection signatures (Figure 3). An unexpected result was obtained for the *COX* genes: they show adaptive signatures across all types of analyses at the same level as more variable *ND* genes despite the greatest conservatism.

Remarkably, the largest number of genes under selection was found in *P. schaposchnikowi*, *Ellobius* species, and *L. mandarinus* (Figure 3). Among them, *P. schaposchnikowi* is the oldest subterranean lineage within the subfamily, representing the first wave of species radiation within Arvicolinae [73] and the earliest split within the subfamily [74,75]. *Ellobius* species (tribe Ellobiusini) and *L. mandarinus* (tribe Arvicolini) are significantly younger and both represent the latest most diverse radiation wave of the Arvicolinae subfamily. Thus, there is no correlation between the number of revealed genes with selection signatures and the evolutionary age of the lineage. For instance, lineages with a similar evolutionary age, such as the *Ellobius* species *H. fertilis*, dramatically differ in selective pressures in mitochondrial genes. While for *Ellobius* species we observe signatures of selection in almost all mitochondrial protein-coding genes, in *H. fertilis* we found only three sites under positive selection in two genes. The same phenomenon is observed while comparing similar-in-age subterranean species of the Arvicolini tribe: *T. subterraneus*, *T. daghestanicus,* and *L. mandarinus*. While *Terricola* species do not show any signatures of selection, *L. mandarinus* in contrast, is the second one after the mole voles in the number of such signatures in mitochondrial genes.

In total, we have found the highest number of selection signatures related to the subterranean lifestyle in the *Ellobius* species, which are younger than the oldest representative of the subfamily, subterranean *P. schaposchnikowi*, by ca. 2 Ma. However, if viewed in light of the degree of specialization to the subterranean lifestyle, the data obtained seem to be quite logical. The most stressful challenges faced by subterranean rodents are hypoxic/hypercapnic conditions and overheating in the closed burrow system [2]. While both mole voles and long-claw voles are highly specialized diggers, they occupy different habitats, and their burrows’ characteristics as well as foraging methods are quite distinctive. The typical habitats of *Ellobius* are arid or semi-arid landscapes like steppes, deserts, and grasslands. These rodents populate various soil types, including the compact soils of clay deserts, and create rather stable systems of narrow tunnels to forage underground with geophytes [9,35,40]. Thus, mole voles should cope with issues which other truly subterranean mammals encounter [2]. The latter in turn may lead to the changes we observed in the mitochondrial protein-coding genes. On the contrary, *P. schaposchnikowi* occurs in Caucasian subalpine tall-grass meadows at 1500–2500 m a.s.l. [45,76,77]. Its burrows are excavated in loose humid soils filled with stones which should increase gas diffusion. Importantly, the superficial foraging tunnels of these voles are unusual in that they seem to be oversized being almost twice as wide as one would expect for a rodent of this size ([77]; personal observations of AS). This extra space may prevent overheating, hypoxia, and hypercapnia. Furthermore, the long-clawed mole-vole is strictly herbivorous, at least in summer. During the foraging bouts, the vole sticks out of the hole, picks up plants, and drags them into the burrow to feed in safety ([78,79]; personal observations of AS). Therefore, despite being limited in foraging activity to the vicinity of burrow openings, long-claw voles spend a lot of time out of tunnels. Due to the cold climate, burrow architecture, and feeding behavior, *Prometheomys* may avoid some of the physiological challenges which most subterranean species have to cope with.

We found significant differences in selective pressures on mitochondrial protein-coding genes in *L. mandarinus* and *Terricola* species despite the similar evolutionary age of these taxa. This difference also may reflect unequal levels of energetic and hypoxic stress resulting from specific characteristics of foraging strategies and burrow architecture. Mandarin voles feed either on geophytes underground or green parts of plants in the immediate vicinity of the burrow entrance. Foraging tunnels of this species are located at depths of 10–30 cm [80,81], and the direct measurements of gas concentrations, temperature, and humidity confirmed that animals in the burrow should face hypoxia and hypercapnia. European pine voles inhabit various vegetation communities from broad-leaf forests (*T. subterraneus*) to alpine meadows (*T. daghestanicus*) [82]. Similarly to *Ellobius*, *Prometheomys,* and *L. mandarinus*, they use complex networks of underground runways, and exhibit some external traits associated with fossoriality, and thus are usually referred to as subterranean [46,82]. However, the slender physique and nimble habits of *T. subterraneus* (Abramson and Smorkatcheva, unpublished) as well as the characteristics of its tunnel system distinguish this vole from the specialized subterranean rodents. Feeding runways of this species are located in the very surface soil levels (within 5–10 cm) or even just under leaf litter [46]. These runways afford the voles protection from unfavorable weather and predators which explain the tendency of *Terricola* species for slow life history [83,84,85], but their depth is probably too shallow to significantly prevent gas diffusion resulting in hypoxic/hypercapnic conditions inside. Unfortunately, almost nothing is known about the ecology of *T. daghestanicus* which inhabits Caucasian alpine steppes and meadows. The fact that the voles of this species are reported to find shelter among rocks [45] suggests that they are not so strictly subterranean. Our results confirm this fact, so we didn’t find any changes of selective pressures in mitochondrial genes for this species characteristic for other subterranean species. Unfortunately, we do not have enough data about *H. fertilis* digging features and practices to associate it with detected substitutions.

Our results corroborate the hypothesis that the colonization of the subterranean niche has been associated with the shifts in a selective regime of protein-coding mitochondrial DNA. Our data confirm the recent findings obtained by us in the analysis of *CYTB* sequences of Arvicolinae and several studies on mitochondrial genomes of specialized subterranean rodent families [33,67]. Our data indicate the signatures of positive selection on certain sites in the evolution of mitochondrial DNA in Arvicolinae during colonization of subterranean environments. We demonstrate relaxation of selection in most mitochondrial DNA protein-coding genes using *dN/dS* calculation with branch and branch-site models in phylogenetically distant subterranean arvicoline lineages (e.g., *Ellobius* and *Lasiopodomys*). Thus, we observe similar and convergent trends in the adaptive evolution of mitochondrial genome among major subterranean lineages of Arvicolinae. Nevertheless, by performing a standard set of analyses, we found that various mitochondrial genes demonstrated different levels of evolutionary changes. Among the mitochondrial genes, *ATP8* and *CYTB* showed maximal amounts of selection signals across the studied dataset. We also observe a discrepancy between the number of detected genes with signatures of selection and evolutionary age of the lineage. The highest number of selection signatures was found in the *Ellobius* species—relatively phylogenetically young, yet the most specialized subterranean form of Arvicolinae. Our data provide evidence that signatures of selection in individual mitochondrial protein-coding genes associated with the colonization of the subterranean niche may evolve within a rather short evolutionary period.

## 5. Conclusions

Our comparative study shows signatures of relaxation selection in the evolution of mitochondrial DNA in Arvicolinae during colonization of the subterranean environment. We found that the number of selection signatures in mitochondrial genes is independent of the evolutionary age of the lineage but positively correlated with the degree of specialization to the subterranean niche. The data obtained suggests that the signatures of selection in individual mitochondrial protein-coding genes associated with the entering of the subterranean niche may appear within a rather short evolutionary timespan.

## Figures and Tables

**Table 1:** Estimation of ω in ete-toolkit using a branch model. Fg-foreground branch (subterranean species), Bg-background branch (aboveground species). Subterranean species indicated by color on Figure 1. LRT-likelihood ratio test *p*-value for models’ comparison, M0-one-ratio model, b_fee-free-branch model. Only significant results are given in this table; all values can be found in Table S2.

Gene	Fg, ω1	Bg, ω0	Adjusted *p*-Values LRT b_Free and M0
*Arvicolinae* (all species)			
Concatenate genes	0.0946	0.0516	0.0000
*Ellobius* and Group D			
*ATP6*	0.13	0.04	3.43 × 10^−4^
*ATP8*	0.74	0.16	0.0052
*COX1*	0.03	0.01	5.04 × 10^−5^
*COX2*	0.22	0.02	1.17 × 10^−9^
*CYTB*	0.06	0.02	3.74 × 10^−4^
*ND1*	0.06	0.03	0.0125
*ND4*	0.09	0.04	0.0017
*ND4L*	0.18	0.04	0.0051
*P. schaposchnikowi* and Group E			
*COX3*	0.05	0.01	0.0136
*L. mandarinus* and Group B			
*COX3*	0.12	0.03	0.0324
*CYTB*	0.23	0.03	1.70 × 10^−4^

**Table 2:** Estimation of ω using aBSREL. Name-branch of interest, B-optimized branch length, LRT-likelihood ratio test, Test *p*-value corrected for multiple testing.

Group	Gene	Name	B	LRT	Test *p*-Value	ω Distribution over Sites
*Ellobius* and Group D	*COX2*	*E. lutescens*	0.0072	17.9838	0.0001	ω1 = 0.238 (81%) ω2 = 15.4 (19%)
*P. schaposchnikowi* and Group E	*ND5*	*P. schaposchnikowi*	0.0149	4.4686	0.0390	ω1 = 0.395 (89%) ω2 = 16.7 (11%)
*P. schaposchnikowi* and Group F	*ATP8*	*P. schaposchnikowi*	0.1443	6.0817	0.0171	ω1 = 0.0553 (90%) ω2 = ∞ (10%)

**Table 3:** Estimation of natural selection intensity using the RELAX approach. LRT-likelihood ratio test, P-adjusted *p*-value, K-selection intensity parameter. Only significant results are given in this table, all values can be found in Supplementary Table S3.

Gene	LRT	P	K
*Arvicolinae* (all species)			
Concatenate genes	396.5300	0.000	0.5200
*Ellobius* and Group D			
*ATP6*	38.9000	5.785 × 10^−9^	0.1700
*COX1*	24.8820	7.308 × 10^−6^	0.4070
*COX3*	9.0750	0.0175	0.1320
*CYTB*	17.4720	0.0003	0.4816
*ND1*	14.7850	0.0011	0.6414
*ND2*	23.7070	1.232 × 10^−5^	0.3624
*ND3*	7.977	0.0282	0.5980
*ND4*	14.1950	0.0013	0.1628
*P. schaposchnikowi* and Group F			
*COX1*	18.8005	0.0002	0.3875
*COX3*	12.4548	0.0042	0.4027
*ND2*	21.2713	4.788 × 10^−5^	0.1834
*ND4*	30.1175	5.291 × 10^−7^	0.5331
*ND5*	9.9163	0.0147	0.3350
*P. schaposchnikowi* and Group E			
*COX3*	18.5788	0.0002	0.3382
*L. mandarinus* and Group B			
*COX1*	8.8720	0.0319	0.4922
*COX3*	11.1457	0.0101	0.0071
*CYTB*	22.5555	2.652 × 10^−5^	0.3003

**Table 4:** Sites under positive selection found in mitochondrial genes using the MEME approach. N/A—not analyzed, “—”—analysis did not show sites under positive selection. Homologous site found for several species in *COX1* indicated in bold.

Gene	*Ellobius*	*P. schaposchnikowi* and Group F	*P. schaposchnikowi* and Group E	*L. mandarinus*	*H. fertilis*	*Terricola*
*ATP6*	—	—	—	76	—	—
*ATP8*	64	22, 41	—	60	53, 63	—
*COX1*	**434**	—	**434**, 484	—	—	—
*COX2*	—	—	—	218	—	—
*COX3*	—	—	—	—	—	—
*CYTB*	—	296	380	23, 57, 67, 226, 329, 371	82	—
*ND1*	—	—	—	65, 299	—	—
*ND2*	69	83, 199, 281, 320	—	235	N/A	—
*ND3*	—	—	—	20	—	—
*ND4*	22, 169, 189, 256	188	19, 25, 107, 171, 188, 205, 351	155, 350, 441	—	—
*ND4L*	17	—	—	—	—	—
*ND5*	36	59, 441, 491, 511	213, 542, 551, 602, 603	311, 364	N/A	—
*ND6*	111	—	4, 81	—	—	—

## Data Availability

All analyzed mitochondrial genomes available in GenBank, accession numbers given in Supplementary Table S1.

## Supplementary Materials

The following are available online at https://www.mdpi.com/article/10.3390/genes12121945/s1, Table S1: Species and GenBank accession numbers, Table S2: Estimation of ω in ete-toolkit using a branch model, Table S3: Estimation of natural selection intensity using the RELAX approach, Table S4: Identified parallel substitutions in mitochondrial protein-coding genes.

## References

[1] Begall S., Burda H., Schleich C.E., Begall S. (2007). Subterranean Rodents: News from Underground.

[2] Lacey E.A., Lacey E.A., Patton J.L., Cameron G.N. (2000). Life Underground: The Biology of Subterranean Rodents.

[3] Chaline J., Brunet-Lecomte P., Montuire S., Viriot L., Courant F. (1999). Anatomy of the Arvicoline Radiation (Rodentia): Palaeogeographical, Palaeoecological History and Evolutionary Data. Ann. Zool. Fennici..

[4] Martin R.A. (2015). A Preliminary Diversity-Divergence Model for North American Arvicolid Rodents. Palaeobio. Palaeoenv..

[5] Fejfar O., Heinrich W.-D., Kordos L., Maul L.C. (2011). Microtoid Cricetids and the Early History of Arvicolids (Mammalia, Rodentia). Palaeontol. Electron..

[6] Martin R.A. (2003). Biochronology of Latest Miocene through Pleistocene Arvicolid Rodents from the Central Great Plains of North America. Coloq. Paleontol..

[7] Swingle J.K., Foreman E.D. (2009). Home Range Areas and Activity Patterns of Red Tree Voles (*Arborimus longicaudus*) in Western Oregon. Northwest. Sci..

[8] Corn P.S., Bury R.B. (1988). Distribution of the Voles Arborimus Longicaudus and Phenacomys Intermedius in the Central Oregon Cascades. J. Mammal..

[9] Ognev S.I. (1950). Zveri SSSR I Prilezhashhih Stran (The Mammals of the USSR and Adjacent Countries). Vol VII. Gryzuny (Rodentia).

[10] Avise J.C. (1986). Mitochondrial DNA and the Evolutionary Genetics of Higher Animals. Philos. Trans. R. Soc. B.

[11] Irwin D.M., Kocher T.D., Wilson A.C. (1991). Evolution of the Cytochromeb Gene of Mammals. J. Mol. Evol..

[12] Jaarola M., Martínková N., Gündüz İ., Brunhoff C., Zima J., Nadachowski A., Amori G., Bulatova N.S., Chondropoulos B., Fraguedakis-Tsolis S. (2004). Molecular Phylogeny of the Speciose Vole Genus Microtus (Arvicolinae, Rodentia) Inferred from Mitochondrial DNA Sequences. Mol. Phylogenet. Evol..

[13] Ruiz-Pesini E., Mishmar D., Brandon M., Procaccio V., Wallace D.C. (2004). Effects of Purifying and Adaptive Selection on Regional Variation in Human MtDNA. Science.

[14] Bazin E., Glémin S., Galtier N. (2006). Population Size does not Influence Mitochondrial Genetic Diversity in Animals. Science.

[15] Andrews T.D., Jermiin L.S., Easteal S. (1998). Accelerated Evolution of Cytochrome b in Simian Primates: Adaptive Evolution in Concert with Other Mitochondrial Proteins?. J. Mol. Evol..

[16] Adkins R.M., Honeycutt R.L. (1994). Evolution of the Primate Cytochrome c Oxidase Subunit II Gene. J. Mol. Evol..

[17] Dalziel A.C., Moyes C.D., Fredriksson E., Lougheed S.C. (2006). Molecular Evolution of Cytochrome c Oxidase in High-Performance Fish (Teleostei: Scombroidei). J. Mol. Evol..

[18] Da Fonseca R.R., Johnson W.E., O’Brien S.J., Ramos M.J., Antunes A. (2008). The Adaptive Evolution of the Mammalian Mitochondrial Genome. BMC Genom..

[19] Di Rocco F., Parisi G., Zambelli A., Vida-Rioja L. (2006). Rapid Evolution of Cytochrome c Oxidase Subunit II in Camelids (Tylopoda, Camelidae). J. Bioenerg. Biomembr..

[20] Luo Y., Gao W., Gao Y., Tang S., Huang Q., Tan X., Chen J., Huang T. (2008). Mitochondrial Genome Analysis of Ochotona Curzoniae and Implication of Cytochrome c Oxidase in Hypoxic Adaptation. Mitochondrion.

[21] Shen Y.-Y., Liang L., Zhu Z.-H., Zhou W.-P., Irwin D.M., Zhang Y.-P. (2010). Adaptive Evolution of Energy Metabolism Genes and the Origin of Flight in Bats. Proc. Natl. Acad. Sci. USA.

[22] Tomasco I.H., Lessa E.P. (2014). Two Mitochondrial Genes under Episodic Positive Selection in Subterranean Octodontoid Rodents. Gene.

[23] Goldman N., Yang Z. (1994). A Codon-Based Model of Nucleotide Substitution for Protein-Coding DNA Sequences. Mol. Biol. Evol..

[24] Muse S.V., Gaut B.S. (1994). A Likelihood Approach for Comparing Synonymous and Nonsynonymous Nucleotide Substitution Rates, with Application to the Chloroplast Genome. Mol. Biol. Evol..

[25] Anisimova M., Kosiol C. (2009). Investigating Protein-Coding Sequence Evolution with Probabilistic Codon Substitution Models. Mol. Biol. Evol..

[26] Delport W., Scheffler K., Seoighe C. (2009). Models of Coding Sequence Evolution. Brief. Bioinform..

[27] Thomas S.P., Suthers R.A. (1972). The Physiology and Energetics of Bat Flight. J. Exp. Biol..

[28] Maina J.N. (2000). What It Takes to Fly: The Structural and Functional Respiratory Refinements in Birds and Bats. J. Exp. Biol..

[29] Hassanin A., Ropiquet A., Couloux A., Cruaud C. (2009). Evolution of the Mitochondrial Genome in Mammals Living at High Altitude: New Insights from a Study of the Tribe Caprini (Bovidae, Antilopinae). J. Mol. Evol..

[30] Luo Y., Yang X., Gao Y. (2013). Mitochondrial DNA Response to High Altitude: A New Perspective on High-Altitude Adaptation. Mitochondrial DNA.

[31] Wang Y., Shen Y., Feng C., Zhao K., Song Z., Zhang Y., Yang L., He S. (2016). Mitogenomic Perspectives on the Origin of Tibetan Loaches and Their Adaptation to High Altitude. Sci. Rep..

[32] Yu L., Wang X., Ting N., Zhang Y. (2011). Mitogenomic Analysis of Chinese Snub-Nosed Monkeys: Evidence of Positive Selection in NADH Dehydrogenase Genes in High-Altitude Adaptation. Mitochondrion.

[33] Da Silva C.C., Tomasco I.H., Hoffmann F.G., Lessa E.P. (2009). Genes and Ecology: Accelerated Rates of Replacement Substitutions in the Cytochrome b Gene of Subterranean Rodents. Open Evol. J..

[34] Bondareva O.V., Potapova N.A., Konovalov K.A., Petrova T.V., Abramson N.I. (2021). Searching for Signatures of Positive Selection in Cytochrome b Gene Associated with Subterranean Lifestyle in Fast-Evolving Arvicolines (Arvicolinae, Cricetidae, Rodentia). BMC Ecol. Evol..

[35] Gromov I.M., Polyakov I.Y. (1977). Fauna of the USSR. Mammals. Tome 3. Vyp. 8. Voles (Microtinae).

[36] Musser G.G., Carleton M.D. (2005). Superfamily Muroidea. Mammal Species of the World: A Taxonomic and Geographic Reference.

[37] Abramson N.I., Lissovsky A.A., Pavlinov I.Y., Lissovsky A.A. (2012). Subfamily Arvicolinae. The mammals of Russia: A Taxonomic and Geographic Reference.

[38] Lessa E.P., Vassallo A.I., Verzi D.H., Mora M.S. (2008). Evolution of Morphological Adaptations for Digging in Living and Extinct Ctenomyid and Octodontid Rodents. Biol. J. Linn. Soc..

[39] Smorkatcheva A.V., Lukhtanov V.A. (2014). Evolutionary Association between Subterranean Lifestyle and Female Sociality in Rodents. Mamm. Biol..

[40] Shubin I.G., Sludsky A.A. (1978). The mole voles (Ellobius). Mammals of Kazakhstan. Vol. 1 Gerbils, Voles, Altai Zokor.

[41] Gambaryan P.P. (1960). Adaptive Features of the Locomotory Organs in Fossorial Mammals.

[42] Salvioni M. (1988). Home Range and Social Behavior of Three Species of European Pitymys (Mammalia, Rodentia). Behav. Ecol. Sociobiol..

[43] Kurta A. (1995). Mammals of the Great Lakes Region.

[44] Giannoni S.M., Borghi C.E., Martínez-Rica J.P. (1993). Comparing the Burrowing Behaviour of the Iberian Mole Voles (Microtus (Terricola) Lusitanicus, M. (T.) Pyrenaicus and M. (T.) Duodecimcostatus). Mammalia.

[45] Krystufek B., Vohralik V. (2005). Mammals of Turkey and Cyprus: Rodentia I: Sciuridae, Dipodidae, Gliridae, Arvicolinae.

[46] Mironov A.D. (2020). Spatial Organization of Common Pine Vole (Microtus Subterraneus Selys-Longchamps, 1836) Colonies. Amurian Zool. J..

[47] Smorkatcheva A.V. (1999). The Social Organization of the Mandarine Vole, *Lasiopodomys Mandarinus*, during the Reproductive Period. Z. Saugetierkd..

[48] Abramson N.I., Bodrov S.Y., Bondareva O.V., Genelt-Yanovskiy E.A., Petrova T.V. (2021). A Mitochondrial Genome Phylogeny of Voles and Lemmings (Rodentia: Arvicolinae): Evolutionary and Taxonomic Implications. PLoS ONE.

[49] Darling A.C.E., Mau B., Blattner F.R., Perna N.T. (2004). Mauve: Multiple Alignment of Conserved Genomic Sequence with Rearrangements. Genome Res..

[50] Katoh K., Standley D.M. (2013). MAFFT Multiple Sequence Alignment Software Version 7: Improvements in Performance and Usability. Mol. Biol. Evol..

[51] Ronquist F., Teslenko M., van der Mark P., Ayres D.L., Darling A., Höhna S., Larget B., Liu L., Suchard M.A., Huelsenbeck J.P. (2012). MrBayes 3.2: Efficient Bayesian Phylogenetic Inference and Model Choice Across a Large Model Space. Syst. Biol..

[52] Drummond A.J., Rambaut A. (2007). BEAST: Bayesian Evolutionary Analysis by Sampling Trees. BMC Evol. Biol..

[53] Hassanin A., Léger N., Deutsch J. (2005). Evidence for Multiple Reversals of Asymmetric Mutational Constraints during the Evolution of the Mitochondrial Genome of Metazoa, and Consequences for Phylogenetic Inferences. Syst. Biol..

[54] Lobry J.R. (1995). Properties of a General Model of DNA Evolution under No-Strand-Bias Conditions. J. Mol. Evol..

[55] Cock P.J.A., Antao T., Chang J.T., Chapman B.A., Cox C.J., Dalke A., Friedberg I., Hamelryck T., Kauff F., Wilczynski B. (2009). Biopython: Freely Available Python Tools for Computational Molecular Biology and Bioinformatics. Bioinformatics.

[56] R Core Team (2020). European Environment Agency. https://www.eea.europa.eu/data-and-maps/indicators/oxygen-consuming-substances-in-rivers/r-development-core-team-2006.

[57] Yuan F., Nguyen H., Graur D. (2019). ProtParCon: A Framework for Processing Molecular Data and Identifying Parallel and Convergent Amino Acid Replacements. Genes.

[58] Yang Z., Nielsen R., Goldman N., Pedersen A.-M.K. (2000). Codon-Substitution Models for Heterogeneous Selection Pressure at Amino Acid Sites. Genetics.

[59] Yang Z. (2007). PAML 4: Phylogenetic Analysis by Maximum Likelihood. Mol. Biol. Evol..

[60] Smith M.D., Wertheim J.O., Weaver S., Murrell B., Scheffler K., Kosakovsky Pond S.L. (2015). Less Is More: An Adaptive Branch-Site Random Effects Model for Efficient Detection of Episodic Diversifying Selection. Mol. Biol. Evol..

[61] Wertheim J.O., Murrell B., Smith M.D., Kosakovsky Pond S.L., Scheffler K. (2015). RELAX: Detecting Relaxed Selection in a Phylogenetic Framework. Mol. Biol. Evol..

[62] Murrell B., Wertheim J.O., Moola S., Weighill T., Scheffler K., Kosakovsky Pond S.L. (2012). Detecting Individual Sites Subject to Episodic Diversifying Selection. PLoS Genet..

[63] Delport W., Poon A.F.Y., Frost S.D.W., Kosakovsky Pond S.L. (2010). Datamonkey 2010: A Suite of Phylogenetic Analysis Tools for Evolutionary Biology. Bioinformatics.

[64] Pond S.L.K., Muse S.V. (2005). HyPhy: Hypothesis Testing Using Phylogenies. Statistical Methods in Molecular Evolution.

[65] Huerta-Cepas J., Serra F., Bork P. (2016). ETE 3: Reconstruction, Analysis, and Visualization of Phylogenomic Data. Mol. Biol. Evol..

[66] Tomasco I.H., Lessa E.P. (2011). The Evolution of Mitochondrial Genomes in Subterranean Caviomorph Rodents: Adaptation against a Background of Purifying Selection. Mol. Phylogenet. Evol..

[67] Tavares W.C., Seuánez H.N. (2018). Changes in Selection Intensity on the Mitogenome of Subterranean and Fossorial Rodents Respective to Aboveground Species. Mamm. Genome.

[68] Zhang T., Lin G., Nevo E., Yang C., Su J. (2013). Cytochrome b Gene Selection of Subterranean Rodent Gansu Zokor Eospalax Cansus (Rodentia, Spalacidae). Zool. Anz..

[69] Bromham L., Rambaut A., Harvey P.H. (1996). Determinants of Rate Variation in Mammalian DNA Sequence Evolution. J. Mol. Evol..

[70] Martin A.P. (1995). Metabolic Rate and Directional Nucleotide Substitution in Animal Mitochondrial DNA. Mol. Biol. Evol..

[71] Martin A.P., Palumbi S.R. (1993). Body Size, Metabolic Rate, Generation Time, and the Molecular Clock. Proc. Natl. Acad. Sci. USA.

[72] Lopez J.V., Culver M., Stephens J.C., Johnson W.E., O’Brien S.J. (1997). Rates of Nuclear and Cytoplasmic Mitochondrial DNA Sequence Divergence in Mammals. Mol. Biol. Evol..

[73] Abramson N.I., Lebedev V.S., Tesakov A.S., Bannikova A.A. (2009). Supraspecies Relationships in the Subfamily Arvicolinae (Rodentia, Cricetidae): An Unexpected Result of Nuclear Gene Analysis. Mol. Biol..

[74] Galewski T., Tilak M., Sanchez S., Chevret P., Paradis E., Douzery E.J. (2006). The Evolutionary Radiation of Arvicolinae Rodents (Voles and Lemmings): Relative Contribution of Nuclear and Mitochondrial DNA Phylogenies. BMC Evol. Biol..

[75] Steppan S.J., Schenk J.J. (2017). Muroid Rodent Phylogenetics: 900-Species Tree Reveals Increasing Diversification Rates. PLoS ONE.

[76] Vereshchagin N.K. (1959). The Mammals of the Caucasus; A History of the Evolution of the Fauna. (Mlekopitayushchie Kavkaza; Istoriya Formirovaniya Fauny).

[77] Vorontsov N.N. (1966). New Data on the Biology and Taxonomic Position of the Long-Clawed Mole Vole (Prometheomys Schaposchnikovi Satunin, 1901). Zool. Zhurnal.

[78] Gambaryan P.P., Karapetyan V.S., Ayrumyan K.A., Kazaryan K.G., Mezhlumyan S.K. (1957). To the Ecology of the Long-Clawed Mole Vole Prometheomys Schaposchnikovi Sat. Mater. Study Fauna Armen. SSR.

[79] Zimina R.P., Yasny E.V. (1977). Observations on the Ecology of the Long-Clawed Mole Vole. Zool. Zhurnal.

[80] Smorkatcheva A.V., Aksenova T.G., Zorenko T.A. (1990). Ecology of the Chinese Vole *Lasiopodomys Mandarinus* (Rodentia, Cricetidae) in Transbaikalia. Zool. Zhurnal.

[81] Sun H., Zhang Y., Wang B., Li Y., Xu W., Mao R., Wang Z. (2019). Investigation on Oxygen and Carbon Dioxide Fluctuations in *Lasiopodomys Mandarinus* Burrows. Pak. J. Zool.

[82] Aulagnier S., Haffner P., Mitchell-Jones A.J., Moutou F., Zima J. (2018). Mammals of Europe, North. Africa and the Middle East.

[83] Caroli L., Capizzi D., Luiselli L. (2000). Reproductive Strategies and Life-History Traits of the Savi’s Pine Vole, Microtus Savii. Zool. Sci..

[84] Jemioło B. (1983). Reproduction in a Laboratory Colony of the Female Pine Voles, Pitymys Subterraneus. Acta Theriol..

[85] Schröpfer R. (1977). Die Postnatale Entwicklung Der Kleinwühlmaus, Pitymys Subterraneus De Selys-Longchamps, 1836 (Rodentia, Cricetidae). Bonn. Zool. Beitr..

